# DNA methylation governs the dynamic regulation of inflammation by apoptotic cells during efferocytosis

**DOI:** 10.1038/srep42204

**Published:** 2017-02-07

**Authors:** Clare A. Notley, Christine K. Jordan, Jenny L. McGovern, Mark A. Brown, Michael R. Ehrenstein

**Affiliations:** 1 Centre for Rheumatology, Division of Medicine, University College London, WC1E 6JF London, UK

## Abstract

Efficient clearance of apoptotic cells (AC) is pivotal in preventing autoimmunity and is a potent immunosuppressive stimulus. However, activation of cells prior to apoptosis abolishes their immunoregulatory properties. Here we show using the antigen-induced model of arthritis that the degree of DNA methylation within AC confers their immunomodulatory plasticity. DNA isolated from resting and activated AC mimicked their respective immune effects. Demethylation of DNA abrogated the protective effect of AC whereas remethylation of AC DNA reversed the effects of activation and restored the ability to inhibit inflammation. Disease suppression or lack thereof was associated with TGFβ and IL-6 production respectively. Apoptotic CD4^+^ T cells from patients with rheumatoid arthritis and systemic lupus erythematosus were demethylated compared to healthy controls and favoured production of IL-6 when cultured with healthy macrophages, in contrast to the TGFβ produced in response to healthy AC. Our data implicate AC DNA methylation as the molecular switch that imprints their regulatory properties.

The delicate balance required to preserve immune homeostasis relies in part on the prompt removal of apoptotic cells (AC) by phagocytes^1^. Disruption to AC clearance can lead to the persistence of apoptotic material which can trigger inflammation and autoimmunity^2^. Macrophages as one of the archetypal professional phagocytes contribute to maintaining an immunosuppressive environment during the engulfment of AC via inhibition of pro-inflammatory cytokines whilst maintaining or increasing production of immunoregulatory cytokines such as TGFβ^3^,^4^,^5^. The potent immunosuppressive effects of AC have spurred the development of therapeutic strategies based on their infusion^6^.

However, engulfment of AC is not always tolerogenic and can instead promote inflammation. For instance, AC generated during infection stimulates rather than inhibits the production of inflammatory cytokines and the induction of Th17 cells^7^. Similarly we reported that intravenous administration of apoptotic dendritic cells (DC) which had been activated with LPS prior to induction of apoptosis were unable to suppress inflammatory arthritis due to a surge in splenic IL-6 from resident splenic cells which blocked TGFβ production, in contrast to apoptotic DC not exposed to LPS^8^. Numerous molecules have been implicated in the recognition and engulfment of AC^1^ and some of these are also thought to impart immunoregulatory properties following efferocytosis^9^. Less is known of the processes by which AC lose their ability to mediate inhibition of inflammation although TLR ligands have been implicated in subverting the immunoregulatory response to AC^7^. A further clue as to how AC limit inflammation has been the observation that DNA CpG motifs expressed on the surface of AC interact with TLR9 receptors to enhance IL-10 secretion by regulatory B cells^10^, though engulfment of the AC was not a feature of this report. DNA can also have pro-inflammatory effects^11^ and therefore we investigated whether alterations in apoptotic DNA could account for the distinct immune effects of resting and activated AC. The ability to generate AC with and without immunosuppressive capacity, the latter through a brief exposure to LPS, afforded the opportunity to interrogate the intrinsic attributes of AC that governs their immunoregulatory capacity^8^. In this study, we show that the level of global DNA methylation within AC determines their ability to induce the production of anti-inflammatory or pro-inflammatory cytokines by phagocytes involved in the clearance of AC.

## Results

### The DNA from activated apoptotic cells (AC) is hypomethylated compared to resting AC

**Figure 1:**
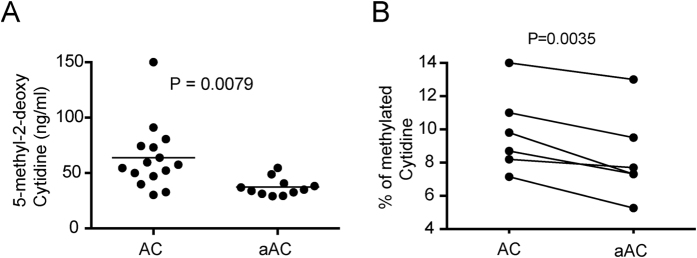
The DNA from LPS-activated AC is hypomethylated compared to unstimulated AC. Bone marrow derived DC were either left unstimulated or activated with LPS overnight followed by induction of apoptosis by addition of etoposide for 5 hr. DNA was isolated from unstimulated apoptotic cells (AC) or activated apoptotic cells (aAC) and global methylation analyzed by ELISA (**A**) or HPLC (**B**). Statistical analysis was performed using an unpaired (**A**) or paired (**B**) t-test.

We have previously shown that LPS activation of dendritic cells (DC) prior to apoptosis induction abolishes their ability to suppress antigen-induced arthritis (AIA) compared to unstimulated apoptotic DC^8^. AC consist of carefully packaged DNA within lipid membranes and blebs that contain all the required surface molecules to allow engulfment and clearance^1^,^12^. We sought to test whether the DNA of the AC mediate their immunoregulatory properties. We further hypothesized that the level of DNA CpG methylation dynamically regulates the balance between inflammation and tolerance. Therefore, we investigated the levels of global methylation of DNA from AC and activated AC. The levels of global methylation were lower in activated AC compared to AC when tested by ELISA (Fig. 1A) and HPLC (Fig. 1B).

### Loss of AC suppressive function after global demethylation of the DNA

**Figure 2:**
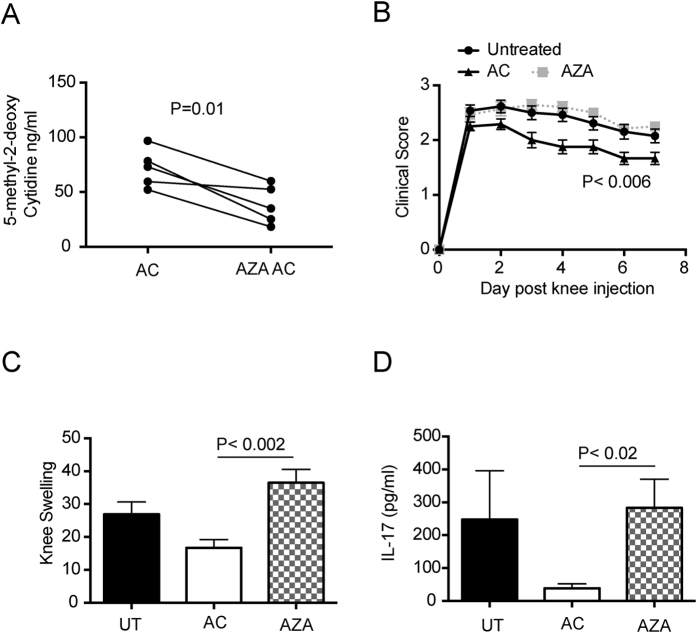
AC with hypomethylated DNA are unable to suppress inflammatory arthritis. DC were left untreated and made apoptotic (AC) or treated with 5-azacytidine and then made apoptotic (AZA AC) and the level of global methylation analyzed by ELISA (**A**). Mice were left untreated (UT) or injected with AC or AZA AC (AZA) at the time of immunization with CFA/mBSA and for a further 2 consecutive days. Arthritis was induced 7 days later and clinical score assessed for a further 7 days (**B**) and knee swelling determined 3 days post knee injections (**C**). 7 days post knee injection, draining lymph nodes were harvested and analyzed for IL-17 production by ELISA (**D**). Graphs shows mean cytokine production ± SEM. Data is pooled from 4 independent experiments with a total of 12 mice per group. Statistical analysis was performed using a t-test (**A**, paired; **C** and **D**, unpaired) or a one-way analysis of variance (ANOVA) followed by Bonferroni post hoc testing for multiple comparisons (**B**).

To study whether changes in global methylation had any functional consequence, we initially took DC and cultured them with 5-azacytidine, an inhibitor of DNA methylation, followed by induction of apoptosis. This led to demethylation of the DNA when compared to untreated cells (Fig. 2A). AC or 5-azacytidine treated AC were then tested for their ability to suppress inflammation by injecting them into mice undergoing induction of AIA. As expected AC were able to lessen the severity of arthritis (Fig. 2B,C) and reduce the production of the pro-inflammatory cytokine IL-17 in the draining lymph nodes (Fig. 2D). In contrast, AZA treated AC with hypomethylated DNA were unable to control the inflammatory arthritis or inhibit IL-17 production in the draining lymph nodes of the inflamed knee (Fig. 2B–D).

**Figure 3:**
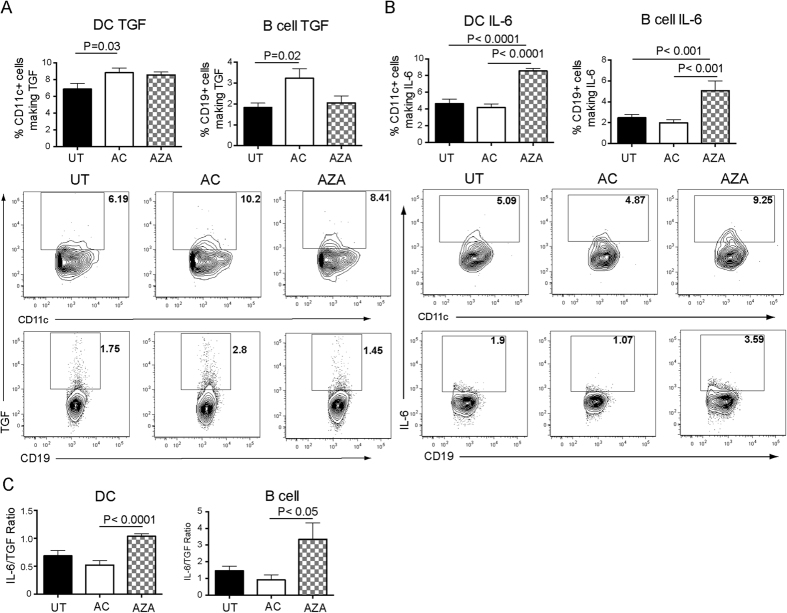
AC with hypomethylated DNA favor production of IL-6 over TGFβ by DC and B cells in mice with inflammatory arthritis. Mice were left untreated (UT) or injected with AC or AZA AC (AZA) at the time of immunization with CFA/mBSA and for a further 2 consecutive days. 14 days after immunization, the percentages of DC (CD11c) and B cells (CD19) in the draining lymph nodes producing TGFβ (**A**) and percentages of DC and B cells in the spleen producing IL-6 (**B**) were determined by flow cytometry. (**C**) Ratio of IL-6 to TGFβ production by DC and B cells. Graphs show mean cytokine production ± SEM of combined data from 3 independent experiments with 9 mice per group. FACS plots show representative data. Statistical analysis was performed using a t-test.

To determine whether the AZA treated AC could alter the balance between the production of TGFβ and IL-6 in the mice with AIA, lymph node and splenic DC and B cells were analyzed for production of TGFβ and IL-6 by flow cytometry. Exposure to 5-azacytidine abolished the increased production of B cell TGFβ induced by AC, though did not significantly change the ability of AC to increase the production of TGFβ by DC (Fig. 3A). 5-azacytidine boosted the production of IL-6 by DC and B cells (Fig. 3B), and increased the IL-6/TGFβ ratio in both these cell types thereby perpetuating inflammation (Fig. 3C). Consistent with previous findings^8^, activated AC stimulated IL- 6 production whereas AC had little impact on this pro-inflammatory cytokine but increased the generation of TGFβ.

### Gain of suppressive function after global re-methylation of the DNA from activated AC

**Figure 4:**
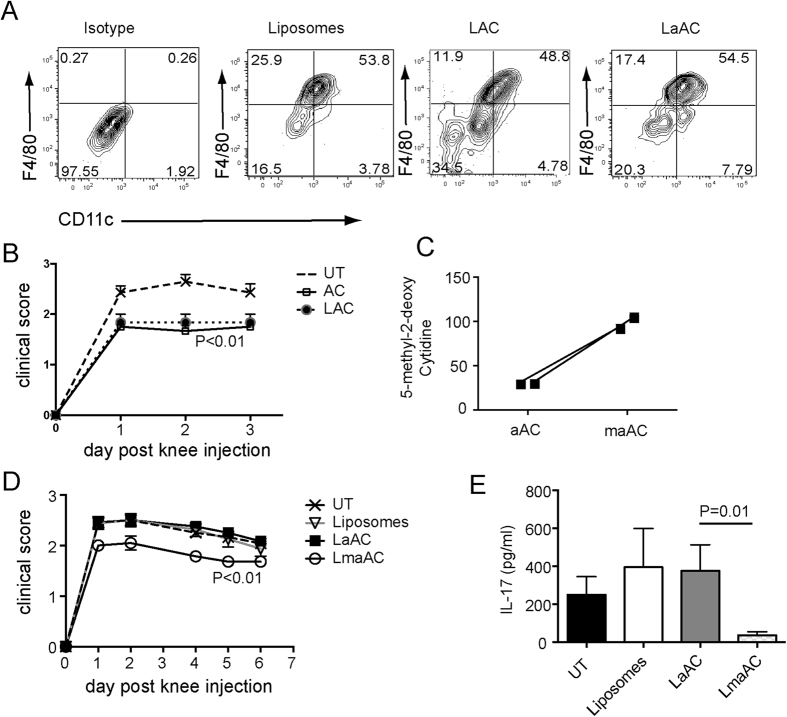
Remethylation of DNA isolated from activated AC restores the ability of the DNA to suppress inflammatory arthritis. (**A**) DNA was isolated from resting or activated AC and loaded into PE-labeled, cationic liposomes. Mice were intravenously injected with liposomes alone or liposomes containing resting AC (LAC), or activated AC DNA (LaAC) and spleens harvested 1 hr later. PE^+^ cells were analyzed for co-staining with F4/80 (macrophages) and CD11c (DC). (**B**) DNA was isolated from resting AC, loaded into liposomes and injected into mice at the time of immunization with CFA/mBSA and for a further two consecutive days. Comparison was made with AC injected into mice at the same time points. Arthritis was induced 7 days later and clinical score assessed for 3 days. Data is pooled from 3 independent experiments, with a total of 8 mice/group. DNA was isolated from activated AC and left untreated (LaAC) or re-methylated with CpG methylase (LmaAC) and loaded into liposomes. (**C**) Prior to liposome loading, global DNA methylation was determined by ELISA. (**D**) Mice were left untreated (UT) or injected with liposomes alone, LaAC or LmaAC at the time of immunization with CFA/mBSA and for a further 2 consecutive days. Arthritis was induced 7 days later and clinical score assessed for a further 7 days. (**E**) 7 days post knee injection, draining lymph nodes were harvested and analyzed for IL-17 production by ELISA. Graph shows mean cytokine production ± SEM. Data is pooled from 4 independent experiments with a total of 12 mice per group. Statistical analysis was performed using one-way analysis of variance (ANOVA) followed by Bonferroni post hoc testing for multiple comparisons (**B**,**D**) or a t-test (**E**).

To further explore whether the level of DNA methylation governs the immunoregulatory properties of AC, DNA from AC was loaded into cationic, PE-labeled liposomes to allow trafficking to the spleen and engulfment by phagocytes. Similar to AC and activated AC^8^, liposomes loaded with DNA from AC or activated AC were engulfed by macrophages and DC 1hr post transfer (Fig. 4A). To examine the inhibitory effects of DNA from AC upon inflammation, liposomes containing AC DNA were transferred at the time of immunization with BSA and for 2 further consecutive days, and arthritis induced 7 days later by intra-articular injection of BSA. Intravenous injection of liposomes containing purified DNA from AC suppressed AIA to the same extent as AC (Fig. 4B). Next the level of methylation of DNA isolated from activated AC was increased using CpG methylase to test whether this would restore their immune suppressing properties. An ELISA was used to confirm efficient methylation of the DNA isolated from activated AC (Fig. 4C) before loading into liposomes. The level of re-methylation achieved was similar to global DNA methylation levels from unmanipulated AC (Fig. 1A). Liposomes were loaded with unmanipulated DNA from activated AC or remethylated DNA from activated AC before injection into mice induced to develop AIA. As expected, liposomes loaded with activated AC DNA (LaAC) had no effect on the severity of arthritis. However liposomes loaded with methylated activated AC DNA (LmaAC) were able to suppress disease (Fig. 4D) and inhibit IL-17 production by the cells in the draining lymph nodes (Fig. 4E).

**Figure 5:**
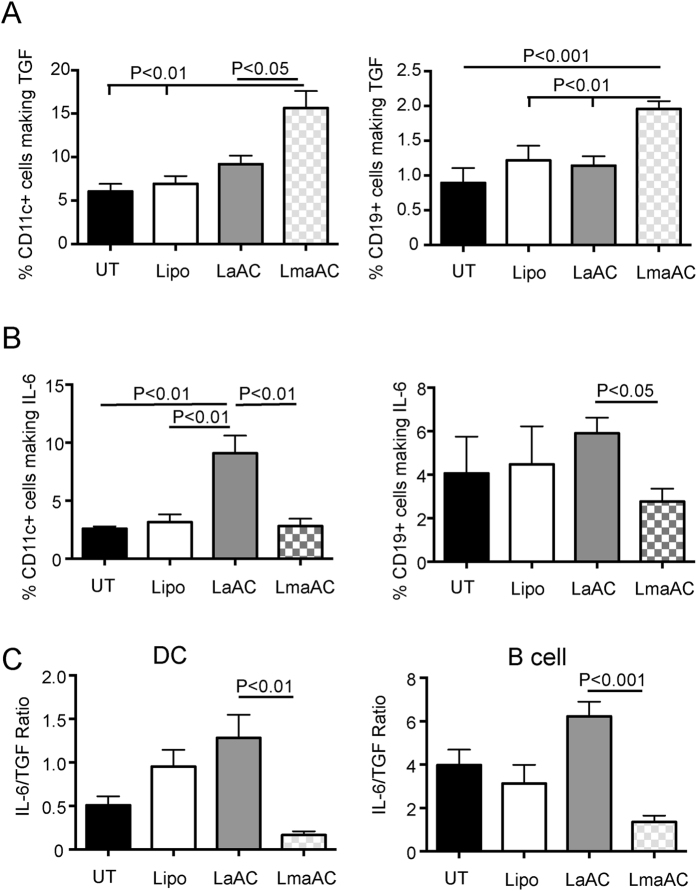
Remethylation of DNA from activated AC reduces IL-6 production and boosts TGFβ by DC and B cells in mice with inflammatory arthritis. Mice were left untreated (UT) or injected with liposomes or liposomes loaded with DNA from activated AC (LaAC) or liposomes loaded with DNA from activated AC that had been re-methylated (LmaAC) at the time of immunization with CFA/mBSA and for a further 2 consecutive days. 14 days after immunization, the percentages of DC (CD11c) and B cells (CD19) in the draining lymph nodes producing TGFβ (**A**) and percentages of DC and B cells in the spleen producing IL-6 (**B**) were determined by flow cytometry. (**C**) Ratio of IL-6 to TGFβ production by DC and B cells. Graphs show mean ± SEM of combined data from 3 independent experiments with 9 mice per group. FACS plots show representative data. Statistical analysis was performed using one-way analysis of variance (ANOVA) followed by Bonferroni post hoc testing for multiple comparisons.

To determine whether remethylation of the hypomethylated DNA from activated AC could alter the balance between TGFβ and IL-6 production by B cells and DC, TGFβ and IL-6 production were investigated in mice with AIA by flow cytometry. Remethylation of the DNA increased the production of TGFβ by DC and B cells in mice (Fig. 5A) whereas the boost in IL-6 production by DNA from activated AC was abolished by remethylation (Fig. 5B) thereby reducing the IL-6/TGFβ ratio (Fig. 5C).

Taken together, our data demonstrate that the DNA isolated from AC is sufficient to regulate an inflammatory response and that its methylation state determines the balance between inflammation and tolerance.

### Apoptotic CD4 T cells from patients with RA and SLE are hypomethylated compared to those from healthy individuals and induce IL-6 production by macrophages

**Figure 6:**
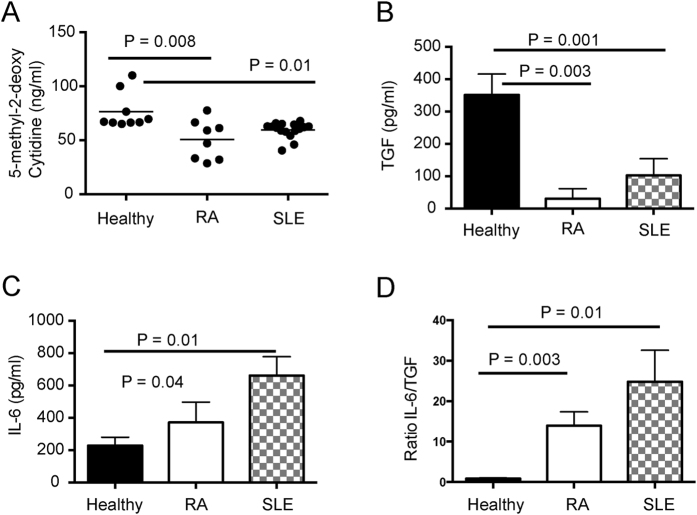
DNA methylation status of human AC is associated with pro- versus anti-inflammatory responses. (**A**) CD4^+^ T cells were isolated from PBMC of healthy controls and patients with RA and SLE and cultured overnight with etoposide to induce apoptosis. DNA was isolated from CD4^+^ AC and digested for the determination of global methylated cytidine levels by ELISA. Graphs show mean ± SEM. Healthy n = 6, RA n = 8, SLE n = 12. (**B**,**C**) The AC generated in (**A**) were cultured with monocyte-derived macrophages from healthy donors for 72 hours. Supernatants were assayed by ELISA for TGFβ and CBA for IL-6. (**D**) Ratio of IL-6 to TGFβ production by monocyte-derived macrophages. Graphs show mean cytokine production ± SEM. Statistical analysis was performed using a t-test.

It is well established that the DNA of T cells from patients with systemic lupus erythematosus (SLE) is hypomethylated^13^,^14^. Moreover, a common feature of SLE is an accumulation of AC^2^. To test the applicability of our results in patients with autoimmune disease, AC were generated from the CD4 T cells of patients with SLE and RA as well as healthy controls. Analysis of the DNA from the AC from patients with RA and SLE confirmed that cytidine residues were hypomethylated compared to their counterparts from healthy controls (Fig. 6A). When AC from healthy controls were cultured with healthy monocyte-derived macrophages, TGFβ production was substantially elevated compared to AC from patients with RA and SLE (Fig. 6B). In contrast, IL-6 production by healthy monocyte-derived macrophages was increased upon engulfment of apoptotic CD4 T cells compared to healthy apoptotic CD4 T cells (Fig. 6C). These changes in cytokine production induced by apoptotic CD4 T cells from RA and SLE patients were reflected by a significant increase in the IL-6/TGFβ ratio from healthy monocyte-derived macrophages (Fig. 6D). This data indicates that the effects of alterations in levels of global methylation of murine AC upon inflammation is also relevant to patients with autoimmune rheumatic disease.

## Discussion

Considerable attention has been paid to the immunoregulatory properties of AC and how they influence inflammation and autoimmunity^1^,^2^. The weight of evidence indicates that AC which are engulfed and safely removed by phagocytic cells are immunoregulatory whereas AC that persist by avoiding engulfment can trigger inflammation. This paradigm has been challenged by observations indicating that infection or LPS activation of cells prior to apoptosis can abrogate their protective properties even when safely cleared by efferocytosis^7^,^8^ although the mechanism underlying the loss of regulatory effects is unknown. Here we demonstrate that the level of AC DNA methylation is responsible for determining an inflammatory or immunoregulatory response induced by AC. The effects of both resting and activated AC can be recapitulated by their DNA which when packaged within liposomes mimics the effects of the AC themselves. The kinetics of the injected liposomes paralleled the AC with respect to engulfment by splenic macrophages and DC. Moreover, we show that manipulation of CpG methylation of AC DNA directly influences the outcome of the immune response. Indeed we can reverse the inflammatory effects of LPS upon cells undergoing apoptosis by remethylation of their DNA resulting in restoration of their immunosuppressive abilities.

The relevance of the murine studies is confirmed through our analysis of apoptotic CD4 T cells from patients with RA and SLE; a similar balance of IL-6 and TGFβ production associates with DNA methylation levels. CD4 T cells from SLE and RA patients are known to possess hypomethylated DNA^13^,^14^, and human CD4 T cell lines can become autoreactive upon exposure of 5-aza-cytidine^15^. From these studies increasing attention has been paid to identification of DNA methylation within particular genes and their participation in triggering or perpetuating autoimmunity. However, our data suggest that there is a role for apoptotic cell DNA as an external ligand controlling inflammatory responses consequent upon AC engulfment rather than acting to repress gene transcription within cells. The fact that DNA can itself be an external stimulus triggering inflammation has long been appreciated although the specific attributes that drive these immunostimulatory properties have not fully emerged^11^. Unmethylated CpG commonly found in microbial DNA^16^ promotes inflammation through the production of IL-6, whereas re-methylation of CpG motifs reverses inflammation^17^. Our studies reveal that activated cells that subsequently undergo apoptosis are pro-inflammatory due to demethylation of their DNA indicating that the degree of methylation is as relevant to the host’s DNA as it is to foreign DNA in triggering or regulating inflammation. Further work will be required to discern the effects of distinct types of activation beyond that of LPS upon cells prior to apoptosis and the signaling pathways triggered following their engulfment.

The mechanisms that sense the global methylation state of the apoptotic DNA and govern the distinct immunological outcomes described here have not been characterized. We have previously shown that at least 80% of AC and activated AC are internalized rather than attached to the surface of the engulfing DC or macrophage^8^. It would be important to determine whether apoptotic DNA also requires internalization to exert its effects or triggers responses through cell surface receptors. There is a plethora of information on pathways triggered by DNA to mediate responses relevant to autoimmunity^18^. TLR9 emerged as the first endosomal DNA sensor relevant for SLE^19^ and indeed its expression on B cells is important for the tolerogenic effects induced by AC clearance^10^. Whilst TLR9 is thought to classically bind unmethylated CpG present in bacterial and viral DNA, thereby inducing an innate immune response, there is mounting evidence that methylated DNA also interacts with TLR9^20^,^21^. Other DNA sensing mechanisms may also be triggered by apoptotic DNA including the DNA binding cytosolic receptors which converge on the ER associated protein STING (stimulator of interferon genes) to induce pro or anti-inflammatory effects^22^,^23^.

In patients with SLE, the production of autoantibodies targeting double stranded DNA and other nuclear antigens are thought to arise as AC undergo secondary necrosis due to clearance deficiencies^2^. However, efforts to enhance the defective removal of AC in diseases such as SLE may not fully restore tolerance and could be potentially dangerous as our data indicate that clearance of activated AC would release IL-6 and not TGFβ. This therapeutic focus should be redirected or complemented by enhancing the methylation of the AC DNA or the signals within the phagocyte that orchestrate these opposing effects of methylated and demethylated DNA. One direct consequence of engulfment of unmethylated DNA is the differentiation of naïve B cells into antibody secreting plasma cells which can contribute to SLE pathogenesis^24^. Our data demonstrated that apoptotic T cells from SLE and RA patients but not healthy individuals not only had hypomethylated DNA but also favoured IL-6 over TGFβ production by macrophages. As well as having a proven role in driving inflammation in RA and SLE^25^, IL-6 enhances the formation of human T follicular helper cells which in turn stimulates the generation of plasmablasts^26^. Compounding these effects, IL-6 promotes hypomethylation of DNA^27^,^28^, which could lead to a cycle of persistent inflammation in patients with autoimmunity. A mouse model of SLE has been characterised that induces disease via the adoptive transfer of hypomethylated CD4^+^ T cells^29^. Taken together with previous work, our data supports a link between hypomethylated DNA within AC, IL-6 production and the development of autoimmunity in both mice and patients.

In summary, this study highlights the importance of DNA methylation in governing the immune response following engulfment of AC. The development of drugs that are capable of modulating DNA methylation may become useful in altering the immunological environment in order to boost or temper inflammation as appropriate. IL-6 blockade, which restored the suppressive properties of activated AC^8^, would be an alternative therapeutic approach made more attractive both by its ability to influence DNA methylation and because IL-6 blockade is already available in the clinic. Our results also offer the prospect of preventing infective episodes from precipitating or exacerbating inflammation through inhibition of pro-inflammatory CpG hypo methylation within apoptotic cells. Immunization with methylated or hypomethylated DNA could be developed as a therapeutic strategy to dynamically control or boost immune responses in a variety of pathological settings including autoimmunity, infection and cancer.

## Materials and Methods

### Mice

C57BL/6 J mice (Jackson Laboratories, CA, USA) were bred and maintained in specific pathogen free facilities under home office guidelines. Mice were used between 8 and 14 weeks of age. All animal experiments and procedures were approved and in the accordance with the guidelines and regulations by the UK Home Office under the UK Animals (Scientific Procedures) Act 1986.

### Preparation of apoptotic cells (AC)

Dendritic cells (DC) were differentiated from the bone marrow of mice by culture for 5 days with 20 ng/ml GM-CSF. On day 5, DC were either left untreated or stimulated overnight with 1 μg/ml LPS (Sigma-Aldrich, MO, USA) followed by extensive washing. DC were then cultured with 10 μM etoposide (Sigma-Aldrich) for 5 hr and the level of apoptosis determined by annexin V and PI staining. Typically both unstimulated AC and activated AC were between 60–75% annexin V positive and 8–11% PI positive. Some DC were incubated from days 2–5 with the GM-CSF in 4 μM 5-azacytidine (Sigma-Aldrich) to inhibit remethylation of CpG sites in the DNA, then induced to undergo apoptosis with etoposide. 5-azacytidine did not alter the percentage of annexin V or PI positive cells after etoposide compared to DC that were untreated before induction of apoptosis with etoposide. Human CD4^+^ T cells were treated with 20 μM etoposide overnight to induce apoptosis.

### Induction of arthritis

Mice were immunized with methylated BSA (mBSA, Sigma-Aldrich) as described previously^30^,^31^. Briefly, 7 days after intradermal immunization with mBSA and CFA, arthritis was induced by intra-articular injection of 200 μg mBSA into the joint cavity. Some mice received 20 × 10^6^ AC or activated AC or demethylated AC i/v. for 3 consecutive days after immunization. Other groups of mice received liposomes either unloaded or loaded with 40 μg of DNA. Clinical score was determined by the degree of limping, where 0 = normal walking, 1 = mild limping, 2 = severe limping and 3 = unable to put weight on leg^31^. Day 7 post intra-articular injection, inguinal draining lymph nodes and spleens were removed and cultured for 72 hr. Supernatants from cultures were tested for IL-17A by ELISA (R&D Systems, Minneapolis, MN). Spleen cells and lymph nodes were also analyzed for the production of TGFβ and IL-6 by flow cytometry.

### Flow Cytometry and ELISA for cytokines

Cells required for intracellular cytokine staining were cultured for 4–6 hr in RPMI containing PMA (Sigma-Aldrich) and ionomycin (Sigma-Aldrich). Golgi-Stop (BD Biosciences) was added to the culture for the last 4hr. Cells were surface stained with anti-CD19-AF700 or V450 (BioLegend, CA, USA), anti-TGFβ-PE (R&D Systems) and anti-CD11c FITC (eBioscience). Cells were then washed and stained with anti-mouse IL-6 FITC or v450 (eBioscience) and analyzed by flow cytometry. Where possible, all gates were set using isotype control antibodies or fluorescence minus one (FMO). All samples were run on the LSR Fortessa and analyzed using FlowJo software (Tree Star, OR, USA). IL-6 levels were assayed in supernatants using the IL-6 CBA Flex kit and buffer set (mouse and human; BD Biosciences) and analyzed using the FACS Verse. TGFβ1 was assayed by ELISA (R&D Systems) after sample acidification.

### DNA Methylation Analysis by ELISA and HPLC

DNA was isolated from murine AC, activated AC and human apoptotic CD4 T cells using the Wizard DNA isolation kit (Promega, WI, USA). The quantity and quality of DNA was assessed by nanodrop. 25 μg of DNA was placed at 100 °C for 3 min and then on ice for 1 min to denature the double helix. The DNA was digested overnight at 37 °C using nuclease P1 (Sigma-Aldrich), in 100 mM ZnCl_2_ and 30 mM sodium acetate. The DNA was then treated with alkaline phosphatase at 37 °C for 2 hr followed by incubation at 100 °C for 10 min and then left on ice before use. The amount of DNA methylation was determined by measurement of the levels of 5-methyl-2-deoxy cytidine (ng/ml) using the DNA methylation ELISA kit (Cayman Chemical Company, MI, USA) following manufacturer’s instructions. Alternatively the level of methylation was quantified using HPLC. The separation of nucleosides was achieved using the Kinetex 5U XB-C18 100 A column (Phenomenex, CA, USA). Deoxycytidine and 5-methyl deoxycytidine were detected using a linear gradient of 0–50% methanol versus 4 mM potassium phosphate, pH 7.0, over 30 min (flow rate, 1 ml/min) and detected at 254 nm. Deoxycytidine (C) and 5-Methyl deoxycytidine (mC) standards (Santa Cruz Biotechnology, Texas, USA) were run as positive controls and doubling dilutions used (75 μg/ml to 3 μg/ml) to calculate the percentage of deoxycytidine that was methylated in samples of AC and activated AC DNA.

### Preparation of DNA-loaded Liposomes

DNA isolated from activated AC was left untreated or incubated at 30 °C with 12 mM S-adenosylmethionine and 4 units of CpG Methylase (Zymo Research, CA, USA) per μg of DNA overnight to methylate CpG sites in the DNA. CpG Methylase was inactivated at 65 °C for 20 min before DNA was ready for loading into liposomes. 40 μg of AC DNA was incubated with 100 μg (total lipid) of DOTAP:DOPE:NBD-PE 100 nm cationic liposomes (Encapsula NanoSciences, TN, USA) at room temperature for 30 min to load the DNA into the liposomes.

### Co-culture of human apoptotic cells and monocyte-derived macrophages

Ficoll-Paque (GE Healthcare) was used to isolate peripheral blood mononuclear cells (PBMC). Monocytes were extracted from the PBMC of a healthy control using CD14^+^ microbeads following the manufacturer’s instructions (Miltenyi-Biotech, CA, USA). Monocytes were cultured at 1 × 10^6^ cells/ml for 7 days in complete RMPI 1640 media (SIGMA) and 40 ng/ml recombinant human M-CSF (PeproTech). CD4^+^ T cells were isolated from the PBMC of healthy controls or patients with systemic lupus erythematosus (SLE) or rheumatoid arthritis (RA) using a CD4^+^ T cell isolation kit, as per manufacturer’s instructions (Miltenyi-Biotech, CA, USA). Apoptosis was induced using etoposide and the cells were thoroughly washed before being cultured alone or in a ratio of 2 AC: 1 macrophage for 72 hours. Supernatants were collected and assayed for IL-6 by CBA or TGFβ by ELISA.

### Patient population

We recruited 15 patients with systemic lupus erythematosus (SLE) all of whom fulfilled the revised classification criteria for disease^32^ and received less than 10 mg/day of prednisolone with either azathioprine or mycophenolate, 8 patients with seropositive (RF or CCP^+^) rheumatoid arthritis (RA) who were treated with conventional DMARDs and less than 10 mg/day of prednisolone and 6 age matched healthy volunteers for this study. All experiments were approved by and in the accordance with the guidelines and regulations of the National Research Ethics Service Committee London appointed by the Health Research Authority. Informed consent was obtained from all patient participants.

### Statistical analysis

All data were analyzed using GraphPad Prism software (CA, USA). Data were analyzed by one-way analysis of variance (ANOVA) followed by Bonferroni post hoc testing for multiple comparisons or using a t-test. The statistical analyses performed are noted with the corresponding data.

## Additional Information

**How to cite this article**: Notley, C. A. *et al*. DNA methylation governs the dynamic regulation of inflammation by apoptotic cells during efferocytosis. *Sci. Rep.* **7**, 42204; doi: 10.1038/srep42204 (2017).

**Publisher's note:** Springer Nature remains neutral with regard to jurisdictional claims in published maps and institutional affiliations.
